# Osteoblasts Growth Behaviour on Bio-Based Calcium Carbonate Aragonite Nanocrystal

**DOI:** 10.1155/2014/215097

**Published:** 2014-03-06

**Authors:** Abdullahi Shafiu Kamba, Zuki Abu Bakar Zakaria

**Affiliations:** ^1^Laboratory of Molecular Biomedicine, Institute of Bioscience, Universiti Putra Malaysia (UPM), 43400 Serdang, Selangor, Malaysia; ^2^Faculty of Veterinary Medicine, Universiti Putra Malaysia (UPM), 43400 Serdang, Selangor, Malaysia

## Abstract

Calcium carbonate (CaCO_3_) nanocrystals derived from cockle shells emerge to present a good concert in bone tissue engineering because of their potential to mimic the composition, structure, and properties of native bone. The aim of this study was to evaluate the biological response of CaCO_3_ nanocrystals on hFOB 1.19 and MC3T3 E-1 osteoblast cells *in vitro*. Cell viability and proliferation were assessed by MTT and BrdU assays, and LDH was measured to determine the effect of CaCO_3_ nanocrystals on cell membrane integrity. Cellular morphology was examined by SEM and fluorescence microscopy. The results showed that CaCO_3_ nanocrystals had no toxic effects to some extent. Cell proliferation, alkaline phosphatase activity, and protein synthesis were enhanced by the nanocrystals when compared to the control. Cellular interactions were improved, as indicated by SEM and fluorescent microscopy. The production of VEGF and TGF-1 was also affected by the CaCO_3_ nanocrystals. Therefore, bio-based CaCO_3_ nanocrystals were shown to stimulate osteoblast differentiation and improve the osteointegration process.

## 1. Introduction

Cockle (*Anadara granosa*) is a marine bivalve molluscs found locally in Malaysia. The shells forms are disposed of as a by-product of the sea/sea food industry, and are treated as waste material, and primarily left at dumpsites to naturally deteriorate [1]. In Malaysia, Cockle shells are among the common natural source of calcium carbonate (CaCO_3_) in the form of aragonite polymorph and composed of 95 to 98% CaCO_3_ [2, 3]. Similar forms of marine shells, such as pearl and nacre, have been extensively investigated for their potential application in bone replacement and regeneration medicine. A previous study has shown that nacre was able to form a tight bond to bone without soft or fibrous tissue intervening, and the material slowly dissolved away [4]. Human *in vitro* studies on osteoblasts have demonstrated the ability of nacre to induce mineralised tissue formation [5, 6]. The potential use of coral minerals was reported in scaffold formation by the system of bone forming and desorbing cells, which remodelled over a long period of time [7]. Coralline minerals are biocompatible and have been used as bone substitutes due to their unique and excellent properties, such as osteoconductive quality and high-speed in bone resorption, and since they do not transmit infectious diseases [5]. Furthermore, coral mineral implants have shown an excellent tolerance, osteointegration, osteoconduction, and progressive resorption with replacement by neoformed bone tissue [5, 6].

Due to the increasing demand in bone graft substitutes for the repair of bone defect and irrigularities, has largely augment in the use of biomaterial for the replacement and treatment of such defect. The use of biomaterial becames necessary due to the hoist complication in traditional methods (orthopedic implants, allografts, and autografts), which may likely be in connection with the risk of infection, improper healing after invasive surgeries, insufficient bone donations to seal gaps completely, while physical and pathological degeneration, representing major concerns in orthopaedic surgery [8]. Many materials have been investigated, but they have not met the exact demand and excellent characteristics needed for tissue engineering and bone regeneration medicine.

Natural bone tissue possesses nanocomposites containing both organic and inorganic components that provide the appropriate physical and biological properties and, as a result, it is crucial for biomaterial to mimic living bone tissue [8]. No single type of material is able to mimic the composition, structure, and properties of native bone. However, consider the role played by biomaterials derived from nature, such as nacre, pearl, and coral, in development and their uses in bone scaffold and the repair of bone defects as cited in the literature [5, 7]. Therefore, the synthesis of nanocomposites from these biomaterials may be the best choice for bone tissue regeneration since they can provide the appropriate matrix environment, integrate desirable biological properties, and provide controlled, sequential delivery of multiple growth factors for different stages of bone tissue regeneration [8].

In this study, we employed two different osteoblast cell lines, hFOB 1.19 human foetal osteoblast bone cells and the MC3T3-E1 mouse osteoblastic cells, which represent both human and animal bone cells, to validate the applicability of CaCO_3_ nanocrystals in both cell types for biomedical relevance. We chose 200 *μ*g/mL concentration of CaCO_3_ because, in our previous study (data not shown), increasing or decreasing the concentration does not affect the viability (i.e., no significant toxicity was observed) up to 400 *μ*g/mL.

## 2. Materials and Methods

### 2.1. Synthesis and Characterisation of Calcium Carbonate Nanocrystals

The synthesis and characterisation of calcium carbonate nanocrystals were carried out using oil-in-water (O/W) microemulsions via higher pressure homogeniser (HPH) as described in our previous report [2]. Transmission electron microscopy (TEM) and field emission scanning electron microscopy (FESEM) were used for the particle characterisation as described in our previous report [2].

### 2.2. Cells Culture

MC3T3-E1 and hFOB 1.19 osteoblast cell lines were purchased from the American Type Culture Collection (ATCC; Manassas, VA, USA). MC3T3 were cultured in DMEM with Earle's salts, L-glutamine, NaHCO_3_, foetal bovine serum, and 100 *μ*g/mL each of penicillin and streptomycin at 37°C in a humidified atmosphere containing 5% CO_2_. The hFOB 1.19 cells were cultured at 34°C in a 5% CO_2_ incubator. The culture medium was a 1 : 1 mixture of Ham's F12 and Medium Dulbecco Modified Eagle's minimal essential medium (DMEM) supplemented with 10% foetal bovine serum and 0.3% G418 (Sigma-Aldrich, USA).

### 2.3. Evaluation of Cell Viability

The MC3T3 and hFOB 1.19 osteoblast cell lines were separately seeded into 96-well plates for 24 hrs. The cells were treated with 200 *μ*g/mL of nanocrystal for 1, 2, and 3 days. The media were removed and replaced with fresh media containing 20 *μ*L of the MTT reagents (Sigma Aldrich) at 37°C for 4 hrs. This culture medium was aspirated, and dimethylsulfoxide (DMSO) was added to the 96-well plates. The optical densities of the solutions were measured at 570 nm with a microplate reader (BioTek, Winooski, VT, USA).

### 2.4. Alkaline Phosphatase Activity (ALP)

The alkaline phosphatase activity was determined according to manufacturer instructions with minor modifications using the ALP activity detection kit (Abcam Inc., MA, USA). Briefly, MC3T3 and hFOB 1.19 osteoblast cells were cultured until they reached 90% confluence. Then, the cells were treated with 200 *μ*g/mL of CaCO_3_ nanocrystals and incubated for another 72 hrs. The cells were washed with PBS and lysed in 0.6 mL of buffer containing 10 mM Tris-HCL (pH 7.5), 0.5 mM MgCl_2_, and 0.1% Triton X-100. The cell lysates were centrifuged at 2,000 g and the soluble portion was used for enzymatic assay. An aqueous solution of 2 mg/mL^−1^ p-nitrophenyl phosphate (PNPP; Zymed Laboratories) was mixed with 0.1 M amino propanol (10 *μ*L/well) in 2 mM MgCl_2_ (100 *μ*L/well) with a pH of 10.4 being prepared. Then, 200 *μ*L of the substrate was added to the 96-well plates and incubated in the dark for about 30 minutes. The enzymatic reaction was stopped by the addition of 0.9 mL/well of 50 mM NaOH. The final product (p-nitrophenol) was quantified at 405 nm using a microplate reader (BioTek, Winooski, VT, USA).

### 2.5. Total Intracellular Protein Synthesis

Total protein synthesis was measured using a commercially available kit (Pierce Chemical) with a slight modification to the Bradford method. The cells were cocultured with 200 *μ*g/mL of CaCO_3_ nanocrystal in media for 1, 3, and 7 days. The supernatant was aspirated and washed with PBS three times and lysed using deionised water and 25 *μ*L 1% Triton X-100. To the 96-well plate, 150 *μ*L of the sample was added to each well followed by 40 *μ*L of the dye. A multichannel pipette was used to mix the samples thoroughly and then they were incubated at room temperature for about 30 minutes. The absorbance was measured using a microplate reader at 595 nm (BioTek, Winooski, VT, USA). The protein content was determined from the standard curve of the absorbance versus known concentrations of albumin run in parallel with the experimental samples.

### 2.6. Calcium Deposition in Extracellular Matrix

The levels of calcium deposition in the acidic supernatants were quantified using a commercially available kit (Abcam Inc., MA, USA) according to manufacturer's protocols. Briefly, after the cells were incubated with the nanocrystal, cell lysates were removed from the substrates, and the remaining extracellular matrix was treated with 0.6 M HCl at room temperature for 24 hours. The absorbance of the samples was measured spectrophotometrically at 575 nm. Calcium levels (*μ*g/L) were calculated from the absorbance of the standard curves versus known calcium concentrations measured in parallel with the experimental samples.

### 2.7. Colorimetric Detection of VEGF Production

VEGF levels in cell supernatants were determined with commercial quantitative sandwich ELISA kits (Abnova HmbH, Heildelberg, Germany). The minimum detectable level was less than 5.0 pg/mL. Triplicate readings (values) were obtained and calibrated against a VEGF standard (7.8–500 pg/mL). When the stop solution was added and colour changed from blue to yellow, the intensity of the colour was measured at 450 nm.

### 2.8. Colorimetric Detection of TGF *β*1 Production

A commercially available enzyme-linked immunoassay ELISA kit (Abnova HmbH, Heildelberg, Germany) was used for the assay. Briefly, cell supernatants were collected and the assay was performed according to the manufacturer's instructions. The enzymatic reactions were stopped by the addition of a sulphuric acid solution and the colour change was measured at a wavelength of 450 nm.

### 2.9. Genotoxicity Assay (BrdU) Assay

Cell genotoxicity was analysed based on the incorporation of BrdU (5-bromo-2′-deoxyuridine) into the synthesised DNA during cell proliferation. MC3T3 and hFOB 1.19 osteoblast cells were seeded into 96-well plates at a density of 1 × 10^5^ cells per well and incubated for 24 hrs. The cells were then washed with PBS and treated with either 200 *μ*L/mL of the CaCO_3_ nanocrystals suspension or the control was treated with 0.1% DMSO. After treatment the cells were subjected to a 5-bromo-2-deoxyuridine (BrdU) labelling assay according to the manufacturer's protocols (Roche Diagnostics GmbH, Mannheim, Germany). The absorbance was measured at 550 nm using a microplate reader (BioTek, Winooski, VT, USA) and the data was presented as the mean ± S.D.

### 2.10. Study of Cells Morphology by Scanning Electron Microscopy (SEM)

MC3T3 and hFOB 1.19 osteoblast cells were placed in 24-well plates at a seeding density of 2 × 10^5^ cells per well. After they reached 80% confluence, they were treated with a suspension of calcium carbonate nanocrystals and incubated in CO_2_ at 37°C for 7 days. The cells were washed twice with PBS (pH 7.4) and centrifuged for 10 min at 3000 rpm. The pellets were fixed in 4% (v/v) glutaraldehyde in 0.1 M cacodylate buffer (pH 7.4) for 4 hrs at 4°C. The fixed cells were washed in three changes of sodium cacodylate buffer for 10 minutes each and postfixed in 1% osmium tetroxide at 4°C. The samples were then washed in three changes of sodium cacodylate buffer (pH 7.4) for 10 minutes each, dehydrated in ascending grades of acetone (35%, 50%, 75%, 95%, and 100%), and brought to critical point drying for thirty minutes. The cells were affixed to a metal SEM stub and sputter-coated in gold by using an SEM coating unit (E5100 Polaron, UK). The coated specimens were viewed using SEM (JOEL 64000, Japan) at an accelerating voltage of 25 KV.

All statistical analyses were performed using Minitab statistical software (Minitab Inc, State College, PA, USA) and Origin 8. Treatment effects were determined using one-way analysis of variance followed by Turkey's post hoc analysis. A value of *P* < 0.05 was considered significant unless indicated otherwise.

## 3. Results and Discussion

### 3.1. Characterisation of CaCO_**3**_ Nanocrystals

**Figure 1:**
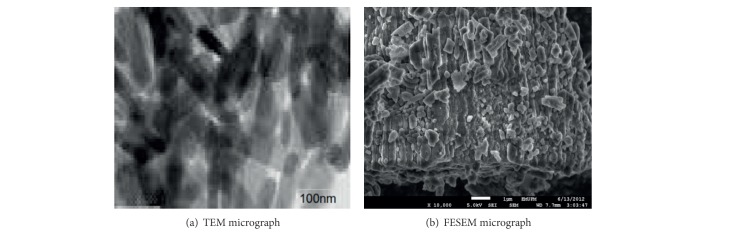
TEM and FESEM micrographs of synthesized calcium carbonate nanocrystals.

Calcium carbonate nanocrystals were fully characterised in our previous published article [2]. As indicated by the transmission electron microscopy (TEM) and field emission scanning electron microscopy (FESEM) micrographs in Figures 1(a) and 1(b), the synthesised calcium carbonate nanocrystals had a perfect rod-shaped morphology, with a uniformly distributed average size of 35–60 nm.

**Figure 2:**
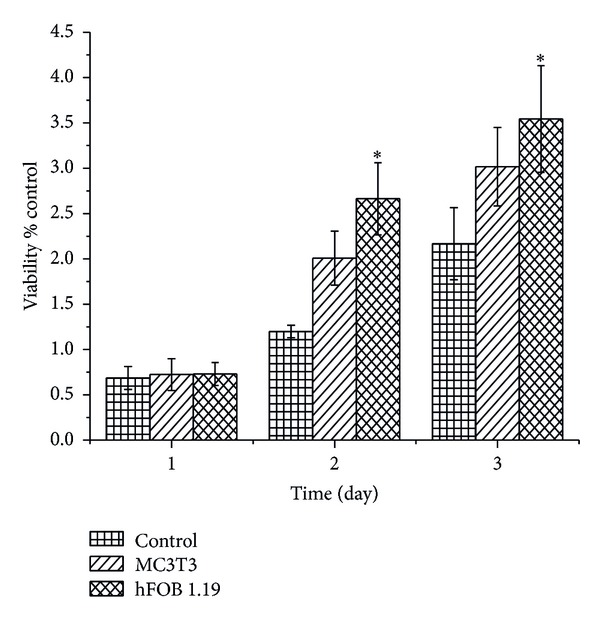
Viability (MTT) in response to calcium carbonate nanocrystals. *Means with different superscripts are statistically significant *P* < 0.05 compared to MC 3T3.

The response of the MC3T3 E-1 and hFOB 1.19 osteoblast cells was evaluated by examining the toxicity of CaCO_3_ nanocrystals during the studies.  Figure 2 shows the proliferation of the cells with longer culturing times. We observed that the hFOB 1.19 osteoblast cells proliferated more when compared to the MC3T3 cells during the incubation periods. Therefore, the nanocrystals affected the metabolic activity of the cells by enhancing the growth and differentiation of the two different osteoblast cell lines. The increase in cell number (optical density; OD) for both hFOB 1.19 and MC3T3 cells was not significant at day 1 when compared to the control. However, there was an abrupt and significant increase (*P* < 0.05) in the number of hFOB 1.19 cells on days 2 and 3 when compared to MC3T3 cells. Therefore, this viability study may indicate that calcium carbonate nanocrystals derived from cockle shells can facilitate osteoblast proliferation, differentiation, and adhesion. Similar research was conducted using pearl shell nanograde powder where the researchers observed increased in cell viability without apparent toxicity [9]. Increase in osteoblast proliferation was observed after being treated with HA-coated magnetic nanoparticles compared to uncoated HA and after 5 days of treatment [10].

### 3.2. Alkaline Phosphatase Activity

**Figure 3:**
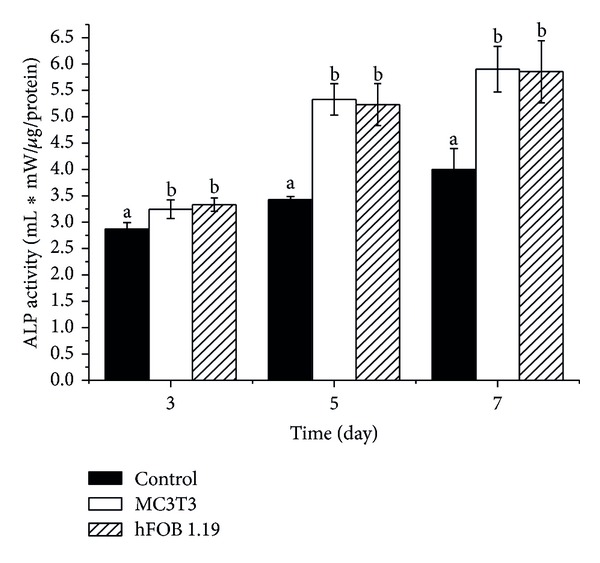
Influence of CaCO_3_ nanocrystals surfaces on ALP synthesis. ^a,b^Means with different superscripts are statistically significant *P* < 0.05 compared to respective group within days.

Alkaline phosphatase activity was examined to determine cellular differentiation after treatment with CaCO_3_ nanocrystals for time duration indicated in Figure 3. The ALP activity of the cells increased significantly over time when compared to control; however, no significant differences were detected between the two cell types (*P* > 0.05). This result indicates that the osteogenic ALP activity was enhanced by the CaCO_3_ nanocrystals. We observed that the increase in osteogenic ALP activity may promote differentiation of mesenchymal stem cells MSCs [9]. Alkaline phosphatase activity is an indicator or bone formation biomarker that signifies bone mineralisation by initiating and promoting the formation of HA in osteoblast matrix vesicles, thereby releasing it into the extracellular matrix and enhancing osteoblast cell differentiation [10].

#### 3.2.1. Total Protein Synthesis

**Figure 4:**
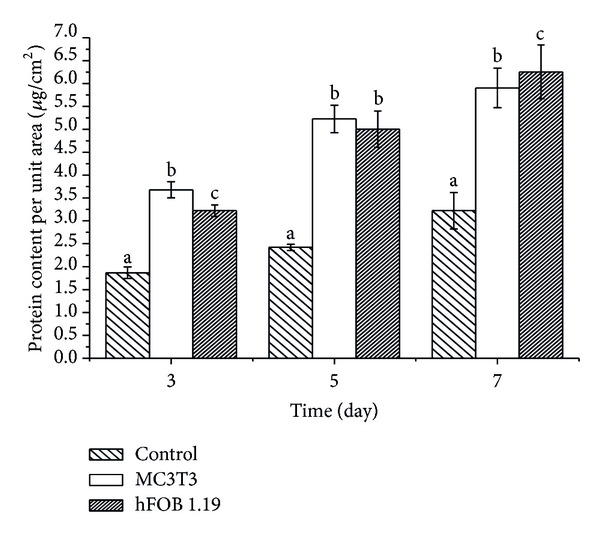
Effect of CaCO_3_ nanocrystals surfaces on protein synthesis. ^a,b,c^Means with different superscripts are statistically significant *P* < 0.05 within days compared to respective group.

Figure 4 shows the expression of proteins synthesised by the cells after being cocultured with nanocrystals for 3, 5, or 7 days. The protein expression by the analysed cells increased in a time-dependent manner as a result of treatment with the CaCO_3_ nanocrystals. Osteoblast MC3T3 cells synthesised more protein when compared to hFOB 1.19 cells by day 3. However, by day 7, the hFOB 1.19 cells increase in total intracellular protein significantly compared to MC3T3. Thus both cells produced significantly higher amounts (*P* < 0.05) of intracellular protein when compared to the control, as shown in Figure 4.

### 3.3. Extracellular Calcium Depositions

**Figure 5:**
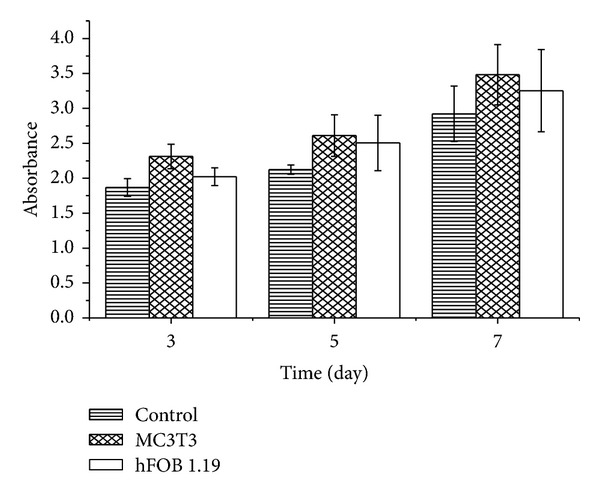
Extracellular calcium deposition in the presence of CaCO_3_ nanocrystals.

Calcium depositions on cultures are one of the most important markers of bone formation and differentiation. Calcium deposition was detected after 3, 5, and 7 days of culture. Osteoblasts cultured with CaCO_3_ nanocrystal exhibit higher deposition of extracellular calcium when compared to the control cells. Moreover, higher calcium deposition was observed in MC3T3 cells when compared to hFOB 1.19 cells, as shown in Figure 5. This result was similar to the ALP activity results and further confirmed the improved osteoblast differentiation resulting from coculture with the CaCO_3_ nanocrystals. Significance increased in extracellular calcium deposition was shown by osteoblast in concentration dependent manner after being cocultured with HA-coated iron oxide nanoparticle for the 21-day culture periods [10].

### 3.4. Lactate Dehydrogenase (LDH) Assay

**Figure 6:**
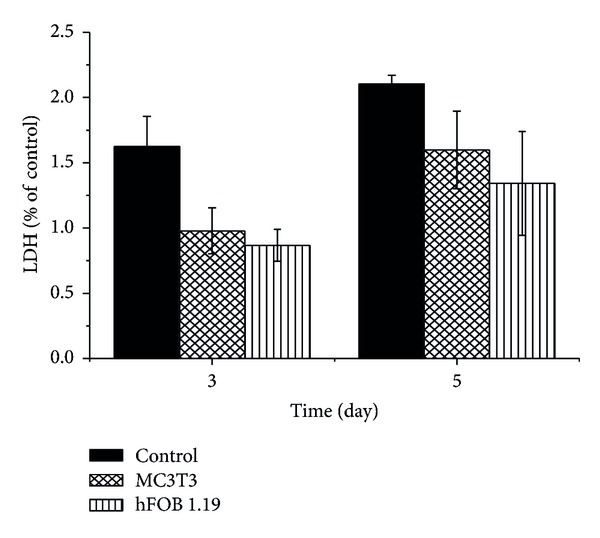
Effect calcium carbonate nanocrystals on LDH release by MC3T3 and hFOB 1.19 osteoblasts cells.

The lactate dehydrogenase (LDH) assay is a convenient method for determining cellular membrane integrity (membrane damage). According to Figure 6, exposure of MC3T3 and hFOB 1.19 osteoblast cells to calcium carbonate nanocrystals caused minimal LDH leakage. This was observed only on day 5 of carbonate nanocrystals administration. While LDH release from hFOB 1.19 cells was higher when compared to MC3T3 cells, the optical densities of both cells were lower when compared to the control cells. Therefore, cytosolic enzymes can leak into extracellular fluids only when the cell membrane integrity is lost [11]. Consequently, the LDH release analysed indicates a minor membrane damage observed due to the effect of CaCO_3_ nanocrystals.

### 3.5. BrdU (Cell Proliferation Assays)

The BrdU assay is based on the ability of proliferating cells to incorporate the BrdU reagent into their DNA as they add thymidine during DNA replication and synthesis. As such, when DNA is damaged, BrdU cannot be incorporated into the DNA during replication, thus indicating a genotoxic effect [12].

**Figure 7:**
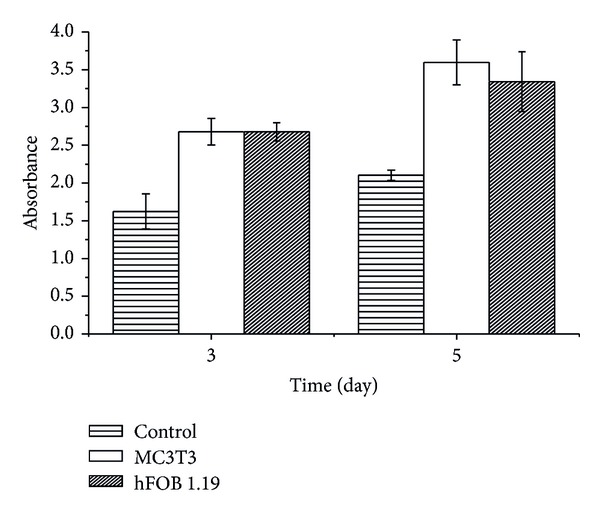
BrdU assays (genotoxicity test), MC3T3 and hFOB 1.19 osteoblast cells treated with CaCO_3_ nanocrystals.

The results shown in Figure 7 suggest a proliferation of both MC3T3 and hFOB 1.19 cells after being cocultured with CaCO_3_ nanocrystal for 5 days. The cellular proliferation observed within the stated time was not significant when compared between the MC3T3 and hFOB 1.19 cells. However, a slight decrease was observed for hFOB 1.19 cells with the highest inhibition rate at 5 days. Furthermore, Figure 7 indicate a minor decrease in cell proliferation due to the the percentage of BrdU incorporated in both MC3T3 and hFOB 1.19 osteoblast cells. This observation in cells decrease was only apparent and significant in hFOB 1.19 cells, where both the cells were significantly higher compare to control cells. These results indicate slight DNA damage due to the reduced viability of the cells (Figure 7). Hernández-Ortiz et al. [12] examine genotoxic effect of opal nanoparticles on 3T3 cells which shows less toxicity to DNA and the percentage of incorporated BrdU in 3T3 cells indicated a minor DNA damage of the cells; they further showed that opal nanoparticles did not cause repairable DNA damage to the cells indicating cytocompatibility of opal nanoparticles [12].

### 3.6. VEGF Production

**Figure 8:**
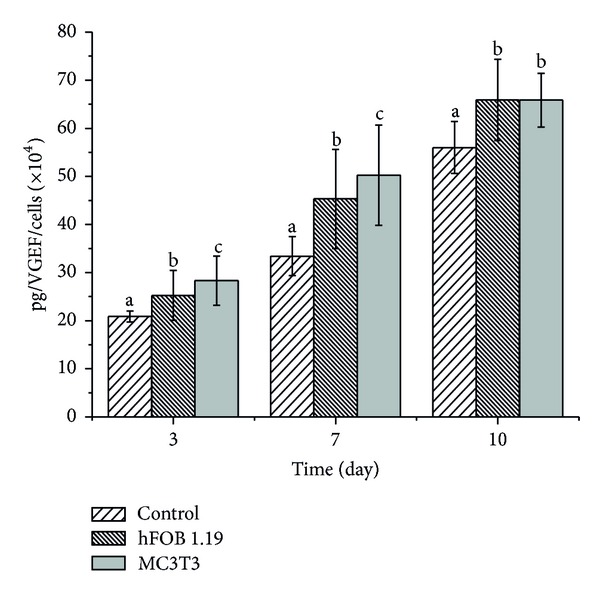
VEGF production in response to CaCO_3_ nanocrystals. ^a,b,c^Means with different superscripts are statistically significant *P* < 0.05 compared to respective group and day.

The study showed that CaCO_3_ nanocrystal increases production of VEGF in the treated cells in time dependent manner. While MC3T3 cells secreted more VEGF when compared to hFOB 1.19 cells at 3 and 7 days, by the last day of treatment the cells show no difference in VEGF production. As a result, the VEGF expression by the two types of cells was significantly different (*P* < 0.05) when compared to the control cells as shown in Figure 8. Osteoblast cells have been reported to produce and secrete VEGF in response to various physiological agents [13, 14].

Recent *in vitro* studies have suggested that VEGF may be directly involved in bone formation [15]. Deckers et al. [15] further reported that VEGF synthesis and receptor expression were established during differentiation processes in osteoblasts cells. Tanase et al. [13] observed that primary human osteoblasts cell migration and differentiation were directly stimulated by VEGF. Also *in vivo* studies proved that VEGF is an important factor for monitoring biologic or pathologic tissue response to an implant or transplant [16–18]. Therefore, VEGF is a well-known powerful proangiogenic growth factor that exerts well-established actions on endothelial cells [13].

### 3.7. TGF-*β*1 Production

**Figure 9:**
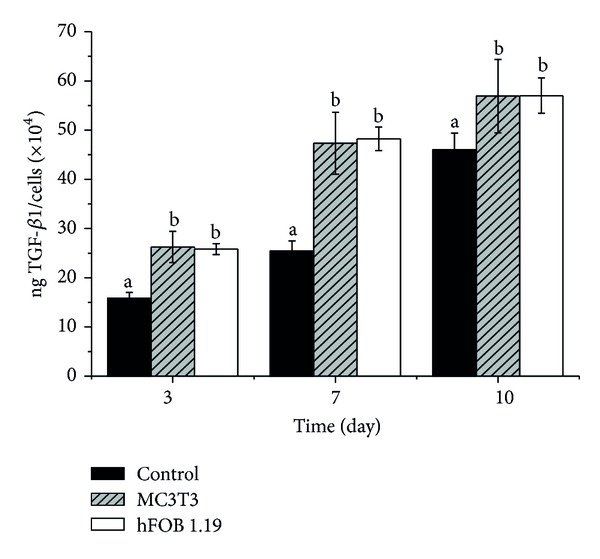
TGF-*β*1 production in response to CaCO_3_ nanocrystals. ^a,b^Means with different superscripts are statistically significant *P* < 0.05 compared to respective group.

The data in Figure 9 shows the production of TGF-*β*1 by the treated osteoblast cells. The results show that TGF-*β*1 synthesised by the cells was not significantly different in either of the treated cells, but that production by CaCO_3_ nanocrystal cocultured cells was significantly different when compared to the control (untreated) cells. The results from *in vitro* and *in vivo* studies suggest that TGF-*β*1 is an important growth factor for bone formation and physiologically upregulates differentiation of osteoblasts [17].

### 3.8. Fluorescence Microscopic Analysis

**Figure 10:**
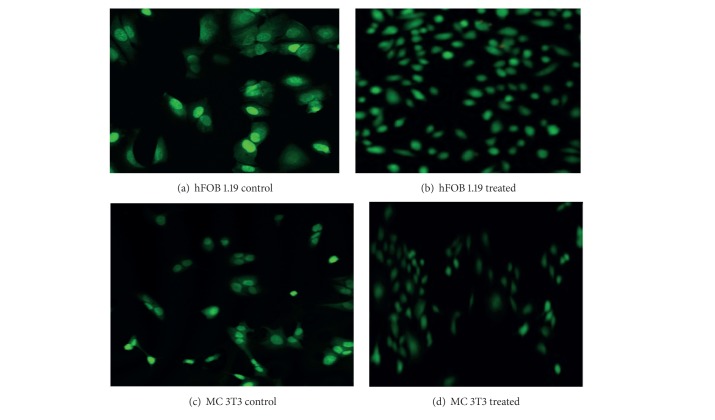
Morphology of MC3T3-E1 and hFOB 1.19 cells attached to CaCO_3_ nanocrystals after 3 days of coculture.

Fluorescence microscopic observation was conducted after 3 days of CaCO_3_ coculture. Figure 9 shows adherent cells represented in Figures 10(a), 10(b), 10(c), and 10(d) for hFOB 1.19 and MC3T3 osteoblast cells, respectively; the morphology of the treated cells was compared to control. The results of the analysed cells indicated a well attached and elongated mophology which are the major sign of viable cells. However, higher numbers of cells were evident in the treated hFOB 1.19 cells when compared to the control cells (untreated hFOB 1.19 Figures 10(a) and 10(b)), wheras MC3T3 cells signifies cells proliferation indicating major sign of viable cells such as formation of cells extension with increase in spreading and elongated morphology. We observed that MC3T3 treated cells have more cells in comparison with the untreated counterpart in Figures 10(c) and 10(d). Moreover, the fluorescence images in all the treated and untreated cells were reported to be viable with no evidence in dead cells.

### 3.9. Morphological Study of MC-3T3 and hFOB1.19 Osteoblast Cells by Scanning Electron Microscopy (SEM)

**Figure 11:**
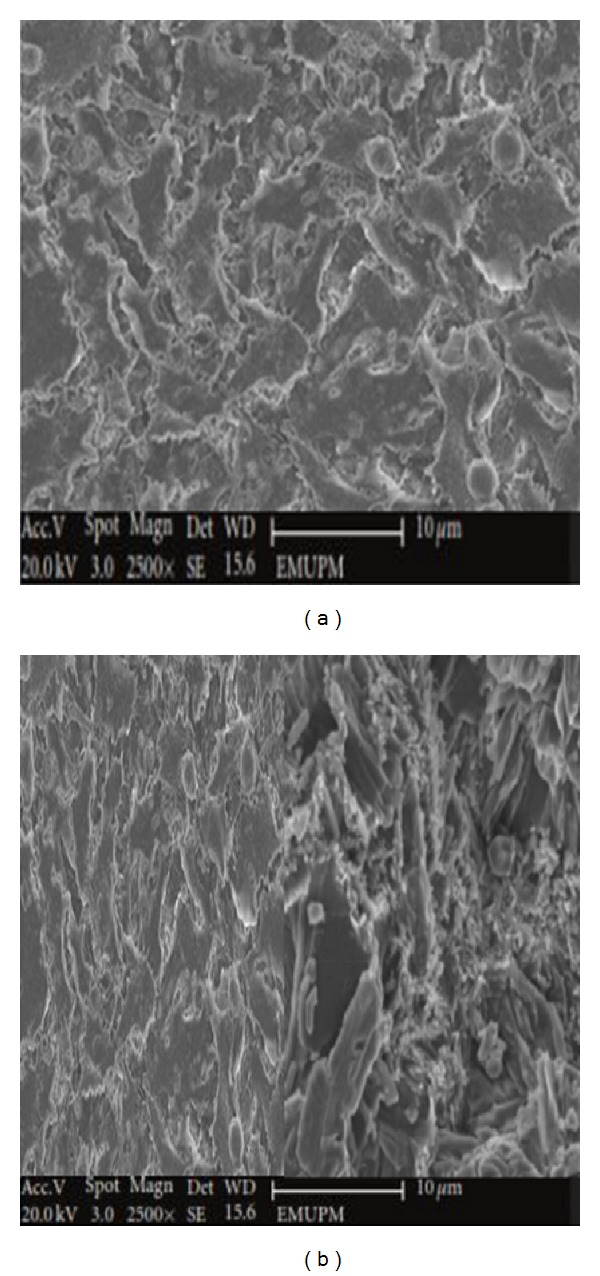
SEM micrograph of (a) control MC3T3 osteoblast cells and CaCO_3_ treated MC3T3 (b).

**Figure 12:**
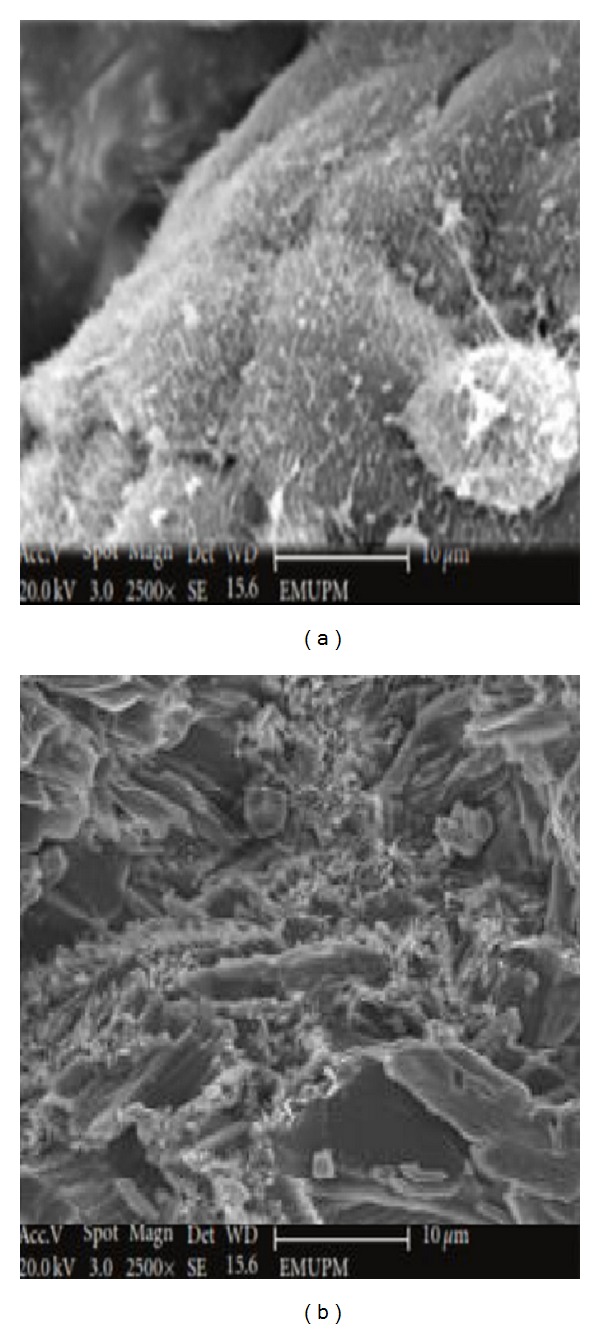
SEM micrograph (a) control hFOB1.19 osteoblast cells and CaCO_3_ treated hFOB 1.19 (b).

The ultrastructural characteristics of MC-3T3 and hFOB1.19 osteoblast cells cocultured on CaCO_3_ nanocrystal surfaces were analysed by scanning electron microscopy (SEM) (Figures 11 and 12). The SEM micrographs of the analysed cells show that they both adhered to the surfaces. Well-attached and proliferating cells were progressively grown on the surface of the calcium carbonate nanocrystals. Densely packed elongated cells have higher spindle like structure, having good attachments to the surfaces of nanocrystal materials. According to Figures 10(b) and 10(d), both cell types expanded well, with cellular membranes and rich cellular plasma. Each of the treated cells exhibited more branched shapes when compared to the controls (Figures 10(a) and 10(c)), while both treated cells grew differently from each other on the surface of nanocrystals. However, the treated MC3T3 cells possessed the aforementioned features when compared to hFOB 1.19 cells. Consequently, this study provides a distinct feature of bio-based CaCO_3_ derived from cockle shells and its ability to improve cells attachment and adhesion. These characteristics make it a promising biomaterial for regeneration medicine.

## 4. Conclusions

This study focused on the mechanistic effect of biogenic calcium carbonate nanocrystals derived from cockle shells on two different osteoblast cell lines. It revealed that CaCO_3_ nanocrystals in the form of aragonite enhanced osteoblast function. It was shown that osteoblast differentiation was also improved, as indicated by the increase in alkaline phosphatase activity, protein synthesis, and extracellular calcium deposition. Additionally, the two osteoblast bone cell lines adhered to the CaCO_3_ nanocrystal surface, as was evidenced by SEM images. Therefore, biogenic CaCO_3_ nanocrystal is a promising and potential biomaterial due to its positive effects and cytocompatibility with osteoblast cell lines. This study may help in providing evidences for Calcium carbonate nanocrystals derived from cockle shells to serve as interesting biomaterials for fabricating bone scaffolds, other orthopaedic applications, and drug delivery systems.
